# Supplementary material for: MetaRibo-Seq measures translation in microbiomes
Source: Nat Commun. 2020 Jun 29;11:3268. doi: 10.1038/s41467-020-17081-z (PMC7324362; doi:10.1038/s41467-020-17081-z)
Supplement: Supplementary file 10 — Supplementary Data 7 [file 41467_2020_17081_MOESM10_ESM.zip › File2/Confidence_VeryHigh_Taxonomy/363979_out.krona.html]

Javascript must be enabled to view this page.

members
magnitude
magnitudeUnassigned
count
unassigned
taxon
rank

363979\_out

6


SRS144362\_contig\_number\_contig-100\_25647.57729
1

2
superkingdom
5

5
1239
phylum

5
class
186801

order
186802
5

1898207
species
2

SRS020233\_contig\_number\_contig-100\_6261.287443SRS144506\_contig\_number\_14753

541000
family
2


SRS020233\_contig\_number\_53152SRS023715\_contig\_number\_23339
2
species
1898205

1950933
species
1

SRS077194\_contig\_number\_22135
